# Assessment of Genetic Diversity in Alfalfa Using DNA Polymorphism Analysis and Statistical Tools

**DOI:** 10.3390/plants13202853

**Published:** 2024-10-11

**Authors:** Cerasela Petolescu, Ioan Sarac, Sorina Popescu, Alina-Maria Tenche-Constantinescu, Irina Petrescu, Dorin Camen, Alina Turc, George Ciprian Fora, Violeta Turcus, Nicolae Marinel Horablaga, Gabriela Gorinoiu, Ganea Mariana, Emilian Onisan

**Affiliations:** 1 Faculty of Engineering and Applied Technologies, University of Life Sciences “King Mihai I” from Timisoara, 119 Calea Aradului Street, 300645 Timisoara, Romania; ceraselapetolescu@usvt.ro (C.P.); ioansarac@usvt.ro (I.S.); sorinapopescu@usvt.ro (S.P.); alinaconstantinescu@usvt.ro (A.-M.T.-C.); irinapetrescu@usvt.ro (I.P.); dorincamen@usvt.ro (D.C.); alina.turc@usvt.ro (A.T.); ciprian.fora@usvt.ro (G.C.F.); 2 Faculty of Medicine, “Vasile Goldis” Western University of Arad, 310045 Arad, Romania; violeta.turcus@uvvg.ro; 3 Faculty of Agriculture, University of Life Sciences “King Mihai I” from Timisoara, 300645 Timisoara, Romania; marinel_horablaga@usvt.ro; 4 Agricultural Research and Development Station Lovrin, 307260 Lovrin, Romania; gabrielagorinoiu@yahoo.com; 5 Faculty of Medicine and Pharmacy, University of Oradea, 10 P-ta 1 December Street, 410073 Oradea, Romania; mganea@uoradea.ro

**Keywords:** alfalfa, genetic diversity, ISSR primers, RAPD markers, molecular breeding strategies

## Abstract

The cultivation of alfalfa is crucial for farmers as it is an excellent forage crop with a high nitrogen-fixing capacity, making it indispensable in crop rotations. Breeding programs face challenges in advancing more rapidly in genetic diversity to achieve a higher heterosis effect and, consequently, greater yield. In this study, we used 30 alfalfa varieties, which were used for molecular analyses by 5 ISSR primers and 13 RAPD primers. The results obtained highlighted the greater efficiency of ISSR primers in identifying genetic diversity. On the other hand, the simultaneous use of ISSR + RAPD allowed for clearer clustering of varieties that enabled more efficiently distinguishing the genetic diversity. The most efficient ISSR primer, A17, generated 31 polymorphic bands, while the most efficient RAPD primer, L-07, generated only 21 bands. Varieties such as “Pastoral” and “F1413-02” exhibited low similarity coefficients (0.39), suggesting their potential for enhancing genetic variability through crossbreeding, thereby increasing the potential of achieving a greater heterosis effect. Conversely, varieties with high similarity coefficients, such as ”Cristal” and “Viking” (0.81) are less suited for this purpose. The correlation between specific markers highlights that using both ISSR and RAPD markers together offers a clear understanding of genetic diversity in alfalfa, aiding in more effective selection for crossbreeding in breeding programs.

## 1. Introduction

Alfalfa (*Medicago sativa* L.) is a perennial crop and the most cultivated and important forage plant grown from the plains to the hilly areas of the world [1]. It is used in the feed rations of many species and categories of animals in the form of green fodder, silage, pellets, or briquettes [2]. Alfalfa is increasingly recognized as a crucial component in sustainable agricultural practices [3]. Its significance is attributed to its contribution to enhancing soil fertility, supporting livestock in diversified farming systems [4,5,6], its high nitrogen-fixation capacity, and its role in mitigating greenhouse gas emissions [7,8,9]. Additionally, due to its extensive root system, alfalfa thrives in arid and semiarid environments with as little as 200 mm of annual rainfall, improving antioxidative defenses and reducing lipid peroxidation, thereby exhibiting resilience to water scarcity [10,11].

Under natural conditions, over 50 tons per hectare of green mass can be obtained, and over 80 tons per hectare under irrigation conditions [12]. However, the very low rates of genetic progress, with a production increase of only 0.30% per year, compared to other forage species, make it difficult for this species to maintain its economic sustainability [13,14].

Alfalfa breeding programs primarily rely on recurrent phenotypic selection and generally focus on developing cultivars with greater disease resistance, higher production capacity, improved nutritional quality, or enhanced tolerance to abiotic and biotic factors [1]. The greatest challenges in alfalfa breeding are related to improving the production capacity. One of the proposals for increasing production capacity was to extend the harvesting period or increase the harvesting intervals [15,16,17]. However, one of the primary strategies in breeding is to maximize heterozygosity or the heterosis effect, particularly in tetraploid alfalfa, as dominant genes seem to play an essential role in increasing production capacity [18,19]. To enhance yield potential, it is crucial to identify and establish the available genetic diversity in the current alfalfa breeding program. This will guide the strategy for selecting crosses to maximize heterosis and improve population proposals, alongside other breeding technologies applicable not only to alfalfa but also to other crops [18,20].

Genetic diversity of the species *Medicago sativa* L. was assessed by numerous researchers from various geographical areas, where alfalfa occupies an important position among the forage legume crops [21,22,23]. A variety of different techniques have emerged to study DNA polymorphisms for the selection of the desired parents in breeding programs, and are widely used for the selection of superior hybrids from a cross population [24,25].

Molecular methods, relying on the PCR technique, have been developed and introduced successfully in the determination of the plant genome genetic profile [26] or in various studies of genetic diversity [27,28].

Some of the most used primers are SSR (Simple Sequence Repeat) primers, which are widely used due to their co-dominant nature [29]. However, they require prior knowledge of target genes. On the other hand, ISSR (Inter-Simple Sequence Repeat) and RAPD (Random Amplified Polymorphic DNA) primers have the advantage of scanning the entire genome without requiring prior knowledge of specific sequences, in this way allowing for the detection of a wider range of genetic variability among the available germplasm [30,31]. Because of this, studies suggest that in the early stages of introducing polymorphic analyses to determine the best crosses, it is preferable to use primers with a broader spectrum of genomic analysis, such as ISSR and RAPD primers, as was the case in our study.

Several authors have used ISSR molecular markers to monitor genetic variability in plants [29,30,32,33,34].

ISSR (Inter-Simple Sequence Repeat) primers are short DNA sequences used in a molecular amplification technique known as ISSR-PCR and used in various studies assessing intra- and intergenotypic variability and in genetic mapping [35]. The advantage of using ISSR primers is that they do not require prior knowledge of the genome and are highly reproducible by amplifying regions of the genome specifically [35,36].

The genetic diversity of some alfalfa populations from the Mediterranean region was assessed with ISSR markers by a research team from Tunis [37]. A variety of different techniques have emerged to study DNA polymorphisms for the selection of the desired parents in breeding programs, and are also widely used for the selection of superior hybrid from a cross population [24,38].

RAPD (Random Amplified Polymorphic DNA) primers are short DNA sequences used in the amplification technique known as RAPD-PCR. Most of these primers are 10 base pairs long, and their advantage is that they can amplify multiple loci across the plant genome without requiring prior knowledge of the plant genome. They are simple, economically feasible, and fast to use. However, they can be very difficult to reproduce, which is a significant disadvantage. The RAPD markers have been successfully applied to assess the genetic variability of the genotypes belonging to the species *Medicago sativa* L. [39,40].

The main objectives our study were to (1) determine the genetic diversity among the 30 Romanian alfalfa varieties, (2) identify the best ISSR and RAPD primers for initial screening of diversity for a more economical approach, and (3) determine the importance of using complementary ISSR and RAPD primers simultaneously in the analysis of diversity.

## 2. Results

The use of ISSR and RAPD primers in studying genetic variability, phylogenetic relationships, and population structure is well recognized in genetic research and plant breeding programs. Our study demonstrates that genomic DNA amplification using ISSR and RAPD primers is an effective method for assessing genetic diversity and intergenotypic variability in alfalfa.

### 2.1. Assessment of Genetic Variability in Alfalfa Using ISSR Markers

To analyze the genetic polymorphism of some Romanian alfalfa genotypes, we tested nine ISSR primers and used five of them: A-12, A-13, A-17, A-21, and UBC-818.

The results obtained confirm the capacity of the A-17 primer to generate polymorphic bands in alfalfa (Table 1).

All five ISSR primers generated polymorphic bands in the 30 alfalfa varieties, with polymorphism rates ranging from 60% to 100%. The mean value of the polymorphism percentage is 78.91%, which indicates a high degree of genetic diversity among the varieties of alfalfa (Table 1).

**Figure 1:**
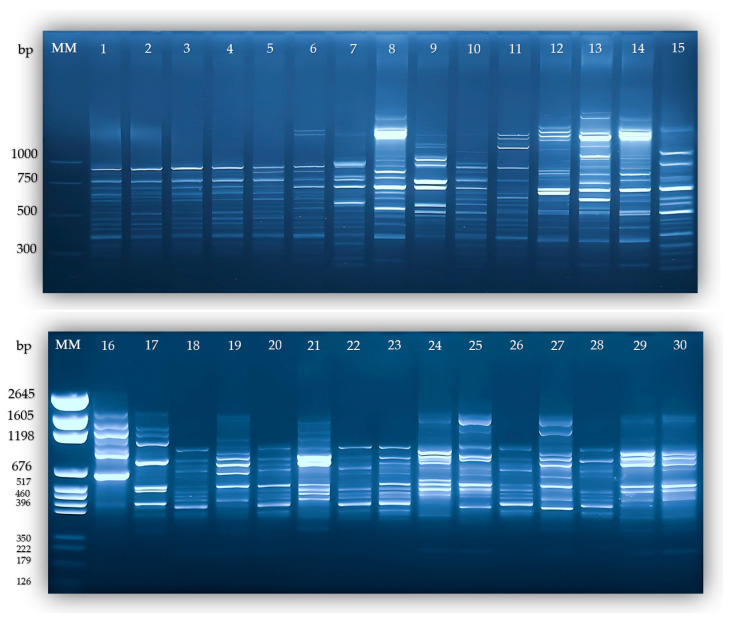
Analysis of agarose gel electrophoresis using A-17 primer: MM—Molecular Marker, 1—Satelit, 2—F1109—99, 3—F105—90, 4—Granat, 5—Cosmina, 6—Sigma, 7—F1615—04, 8—F1206—00, 9—F1306—01, 10—F1822—06, 11—Super, 12—F270—91, 13—F1413—02, 14—F1310—01, 15—F1111—99, 16—Pastoral, 17—Magnat, 18—Alina, 19—Selena, 20—Stolo—13, 21—Mf 42—96, 22—Viking, 23—Cristal, 24—F21991, 25—Coral, 26—Dorina, 27—Saturn, 28—Opal, 29—Venus, 30—F907—97.

According to our results, primer A12 generated 25 bands, and 20 were polymorphic (80.00%); primer A13 generated 27 bands, and 19 were polymorphic (70.37%); primer A21 generated 15 bands, and 9 were polymorphic (60%); primer UBC818 generated 19 bands, and 16 were polymorphic (84.21%); and primer A17 generated 31 bands, with all being polymorphic (100%) (Table 1 and Figure 1).

Regarding the ability to identify the diversity levels among varieties, primer A17 proved to be the most efficient, with a marker index (MI) of 9.17. On the other hand, similar MI values were observed for the other primers, ranging from 3.23 to 3.94 for primers A12, A13, and UBC818. 

**Figure 2:**
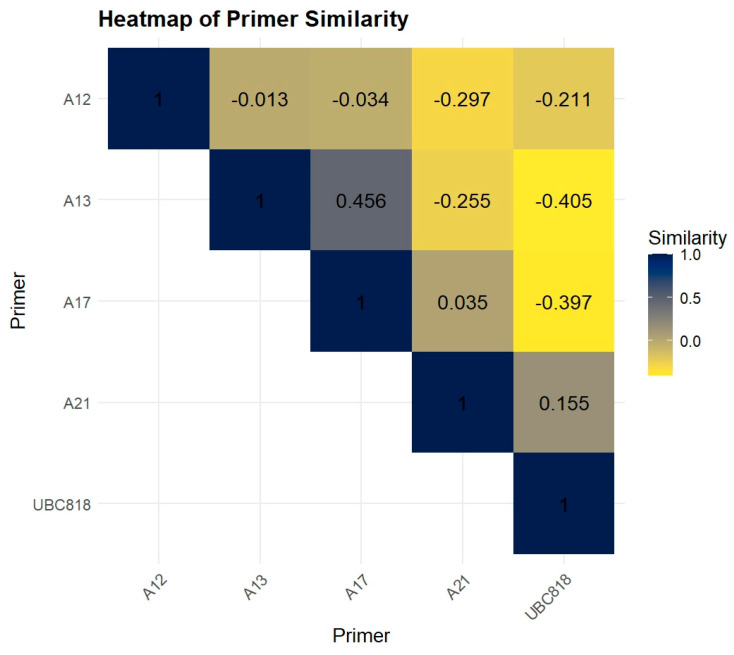
Correlation similarity coefficients values between ISSR primers concerning the genetic similarity of alfalfa genotypes.

The lowest MI value was recorded for primer A21 with a value of 1.41, which also generated the lowest number of bands and the lowest number of polymorphic bands. According to Figure 2, the results of similarity coefficients among ISSR primers did not reveal a very high correlation between any of the primers. The highest similarity was observed between primers A13 and A17 (0.46) (Figure 2).

On the other hand, the similarity coefficients between the other primers generally showed low or negative correlations. From our results, the similarity values between primer A12 and the other primers (A13, A17, A21, and UBC818) are low or slightly negative, ranging from −0.01 to −0.29. This indicates that A12 generates a banding pattern distinct from the other primers.

The most negative correlation was observed between the primer pair A13 and UBC818, with a similarity of −0.40, which suggests when one primer generates a certain band pattern, the other tends to produce a contrasting one (Figure 2).

### 2.2. Assessment of Genetic Variability in Alfalfa Using RAPD Markers

To analyze the genetic diversity of the Romanian germplasm, we tested 16 RAPD primers and used 13 of them. All the 13 RAPD primers generated polymorphic bands, with a rate ranging between 57.14% in the case of the G-03 primer and 100% in the case of the G-19, L-07, L-12, and L-14 primers. The total polymorphism (PIC) generated values between 0.11 in G-03 and 0.27 in L-14.

The marker index (MI) recorded values between 0.25 for the primer G-03 and 4.79 for the L-07 primer, which has the highest capacity of generating polymorphic bands in alfalfa (Table 2).

Following the analysis of genetic diversity in the 30 alfalfa varieties using 13 RAPD primers, these primers amplified segments of the genotypes’ genomes, generating bands with sizes ranging from 170 to 1590 base pairs (bp) (Table 2).

Regarding the number of bands generated, the lowest number, ranging from 7 to 10 bands, was observed with primers G-03, B-07, L-03, and G-06. A moderate number of bands, ranging from 11 to 15, were generated by primers G-16, L-14, G-04, G-18, G-19, G-17, G-10, and L-12.

**Figure 3:**
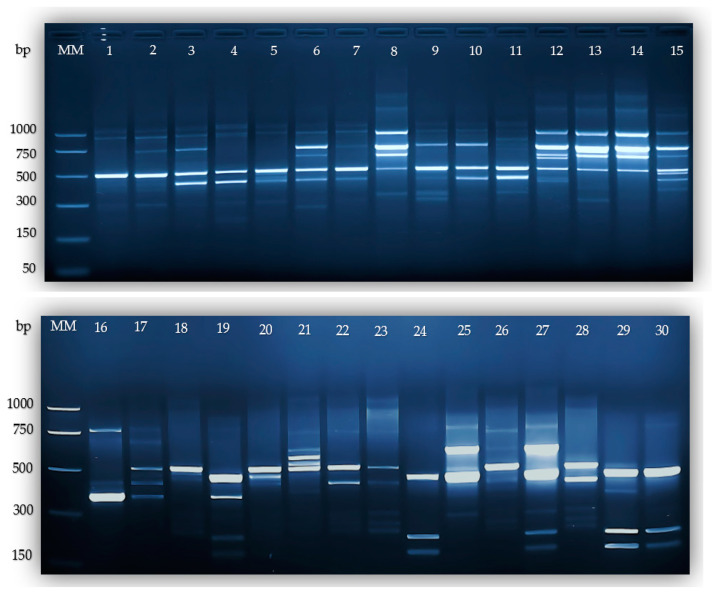
Analysis of agarose gel electrophoresis using L-07 primer: MM—Molecular Marker, 1—Satelit, 2—F1109—99, 3—F105—90, 4—Granat, 5—Cosmina, 6—Sigma, 7—F1615—04, 8—F1206—00, 9—F1306—01, 10—F1822—06, 11—Super, 12—F270—91, 13—F1413—02, 14—F1310—01, 15—F1111—99, 16—Pastoral, 17—Magnat, 18—Alina, 19—Selena, 20—Stolo—13, 21—Mf 42—96, 22—Viking, 23—Cristal, 24—F21991, 25—Coral, 26—Dorina, 27—Saturn, 28—Opal, 29—Venus, 30—F907—97.

The highest number of bands generated was observed for primer L-07, reaching a maximum of 21 bands (Table 2 and Figure 3).

The analysis of polymorphism within the 30 alfalfa varieties indicates a high level of polymorphism, with an average polymorphism percentage of 85.49% across all primer bands generated, signifying a high degree of genetic variability within the analyzed varieties.

By analyzing the polymorphic bands (Pb%) generated by the RAPD primers, the highest percentage is present in primers L-07, G-19, L-12, and L-14. The lowest polymorphism percentage is present in primer G-03, with a Pb% of 57.14%, indicating the lowest efficiency in identifying polymorphism among the alfalfa varieties.

The analysis of the ability of RAPD primers to identify genetic diversity in alfalfa genotypes was expressed by the values of polymorphic information content (PIC) and marker index (MI). PIC values ranged from 0.11 to 0.27, with an average of 0.21, while MI values ranged from 0.25 to 4.79, with an average of 2.10 (Table 2).

The most efficient primer according to our result was L-07, with a PIC value of 0.22 and MI of 4.79, making it the most effective among the primers analyzed (Table 2 and Figure 3).

Other primers with high efficiency include RAPD primer G-19, with a PIC value of 0.26 and MI of 3.46, the most closely related to the primer L-07 efficiency, L-12, with a PIC value of 0.25 and MI of 3.13, and L-14, with a PIC value of 0.27 and MI of 3.01.

On the other hand, some primers exhibited moderate efficiency, such as G-16 with a PIC value of 0.25 and an MI of 1.83, and G-04 with a PIC value of 0.22 and an MI of 1.81.

The lowest efficiency was observed in primers G-03, B-07, and L-03, with MI values ranging from 0.25 to 1.17.

The resolving power index (Rp) ranged from 0.53 to 8.33, with an average of 3.98. The primers with the greatest ability to distinguish among the 30 alfalfa varieties were L-07 and G-19. The highest Rp value, 8.33, was observed with primer L-07 (AGGCGGGAAC), while primer G-19 (GTCAGGGCAA) had an Rp of 6.66. This performance highlights the importance of primer L-07 (AGGCGGGAAC) and G-19 in genetic diversity among varieties of alfalfa.

**Figure 4:**
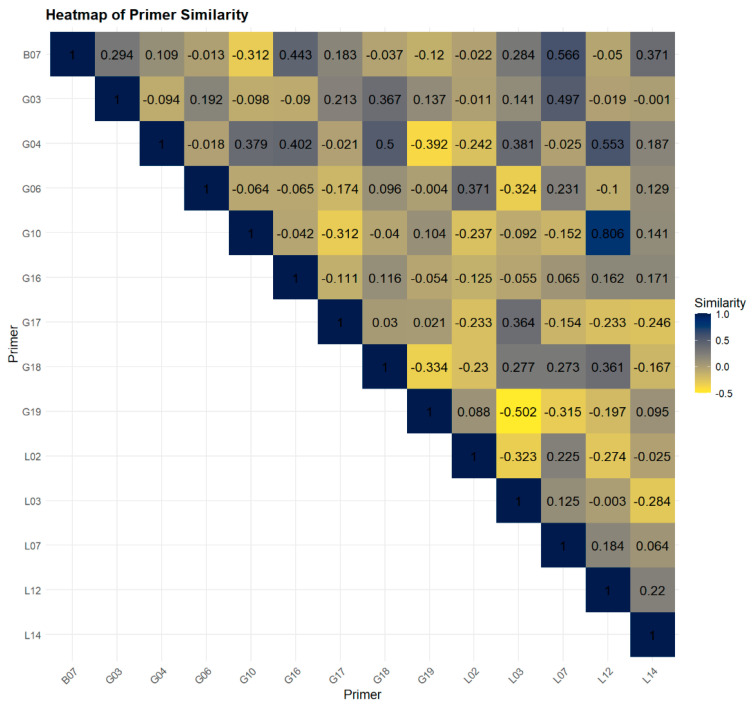
Correlation coefficients values between RAPD primers concerning the genetic similarity of alfalfa genotypes.

According to the correlation coefficient values presented in Figure 4, the RAPD primers showed high levels of similarity in the following pairs: G10 and G19 (0.806), B07 and L07 (0.566), G03 and L07 (0.497), and B07 and G04 (0.443). The high similarity between primers G10 and G19 indicates that they amplify very similar regions of the genome.

Another economical strategy that can be utilized comes from the negative similarity values observed, such as the RAPD primers G19 and G18, which had a negative correlation of −0.502. By combining primers with negative correlations, we managed to increase the efficiency in amplifying different regions of the alfalfa genome, and the effectiveness in detecting genetic diversity among alfalfa varieties could be more easily achieved.

This strategy not only enhances the detection of genetic diversity in alfalfa but also offers an economical approach by selecting groups of primers with negative correlations or eliminating those that are highly similar (Figure 4). Therefore, in such cases, it is more efficient to select a single primer from such a group of primers to avoid amplifying similar regions of the alfalfa genome (Figure 4).

### 2.3. Assessment of Genetic Variability in Alfalfa Using ISSR and RAPD Primers

According to the summary data in Table 3, the ISSR primers generated an average of 23.40 bands per primer, which is significantly higher than the 12.30 bands per primer generated by the RAPD markers. Although the RAPD primers had a higher polymorphism rate of 87.50%, the difference is not statistically significant.

Additionally, the best ISSR primer (A17, Table 1) generated a high number of bands and a discrimination index (MI) of 16.80. In comparison, the most distinct RAPD primer (L-07, Table 2) generated 21 bands with an MI value of 8.33. This highlights the superiority of the ISSR primers used in this study for identifying genetic diversity compared to the RAPD primers (Table 3).

The efficiency index (expressed as the number of polymorphic bands generated per primer) was higher for ISSR primers than for RAPD primers. The range of variation in band size was also significantly greater for ISSR primers (2120 bp) compared to RAPD primers (1590 bp) (Table 3).

Despite using fewer primers (5) than RAPD (13), ISSR demonstrated comparable or superior performance in the efficiency index, which measures the efficiency of primers in producing polymorphic bands. The results indicate that ISSR primers are more efficient per primer used and potentially more resource-effective and economical than RAPD primers in alfalfa varieties. Additionally, the discrimination index is notably higher for ISSR (9.03) compared to RAPD (3.98), highlighting once again ISSR’s greater capacity for distinguishing between different varieties of alfalfa (Table 3).

The results of the analyses conducted using the two categories of primers revealed that, for some genotypes within the studied collection, specific DNA fragments were identified exclusively in certain genotypes (Table 4). These bands can be used as DNA markers for the identification of alfalfa genotypes or for constructing genetic maps. 

Two specific alleles were identified in the Pastoral and Stolo 13 varieties, and in the MF 42-96, F 1111-99, F 1306-01, and F 1206-00 lines, while a single specific band was detected in the Sigma, Cosmina, Granat, Saturn, Dorina, and Coral varieties, and in the F 907-97 and F 1413-02 lines (Table 4).

### 2.4. Clustering of Alfalfa Genotypes Using ISSR and RAPD Primers

**Figure 5:**
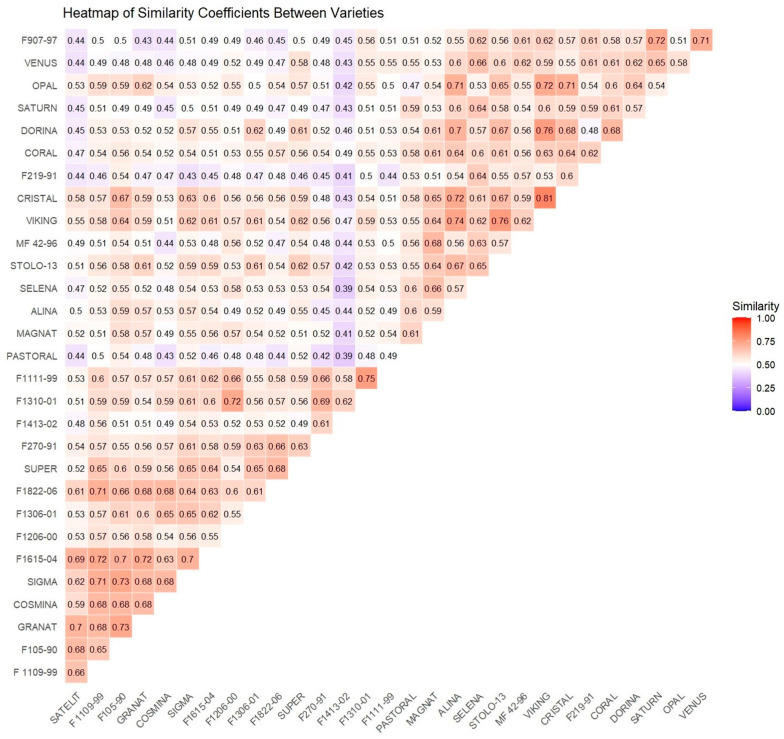
Jaccard similarity heatmap of alfalfa genotypes analyzed with ISSR and RAPD primers.

The molecular analyses resulted in 119 amplified DNA loci from the 5 ISSR primers and 164 amplified DNA loci from the 13 RAPD primers, resulting in a total of 283 amplified DNA loci (Table 1 and Table 2). These analyses and results were instrumental in statistically determining the genetic diversity of the studied varieties using the Jaccard coefficient of similarity (Figure 5). 

**Figure 6:**
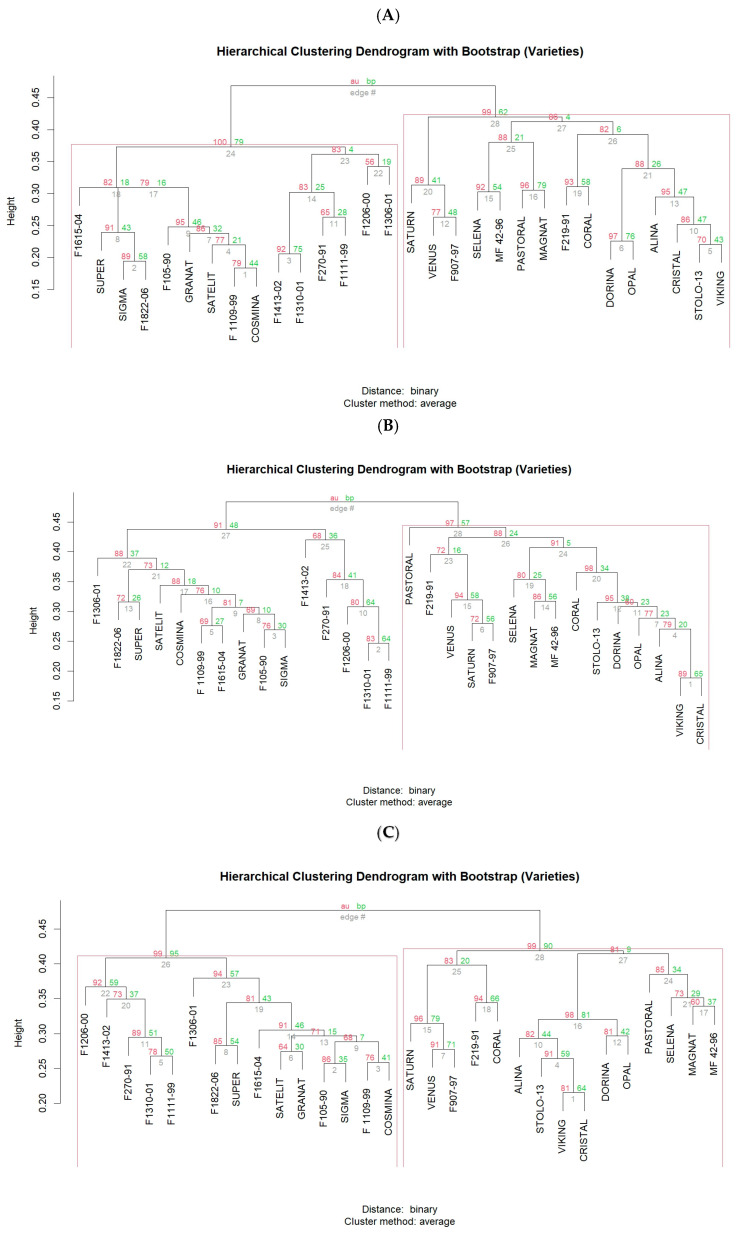
Hierarchical clustering dendrogram with bootstrap values: (**A**) ISSR results, (**B**) RAPD results, (**C**) combined ISSR and RAPD analysis.

The analysis of polymorphisms among the 30 alfalfa varieties revealed very high similarities between certain varieties, such as “Cristal” and “Viking” (0.81) with the highest coefficient. This high similarity was consistently observed across all clustering analyses, including those conducted using ISSR primers and RAPD primers, and the combined ISSR and RAPD analysis (Figure 5 and Figure 6). On the other hand, the lowest similarity value was observed between the varieties “Pastoral” and “F1413-02” (0.39) and “Selena” and “F1413-02” (0.39), indicating that these varieties are very distantly related. This suggests a great potential for crossing these varieties to increase genetic variability in new cultivars (Figure 5 and Figure 6).

The clustering analysis of the 30 varieties was conducted based on their genetic similarity coefficients (Figure 5). Therefore, according to our results, varieties with low similarity coefficients (<0.50) present a higher potential for new crossbreeding strategies aimed at increasing genetic variability. Conversely, varieties within the high similarity group (>0.70) are recommended to be avoided for enhancing heterosis, as they may replicate alleles with limited heterotic effect (Figure 5).

**Figure 7:**
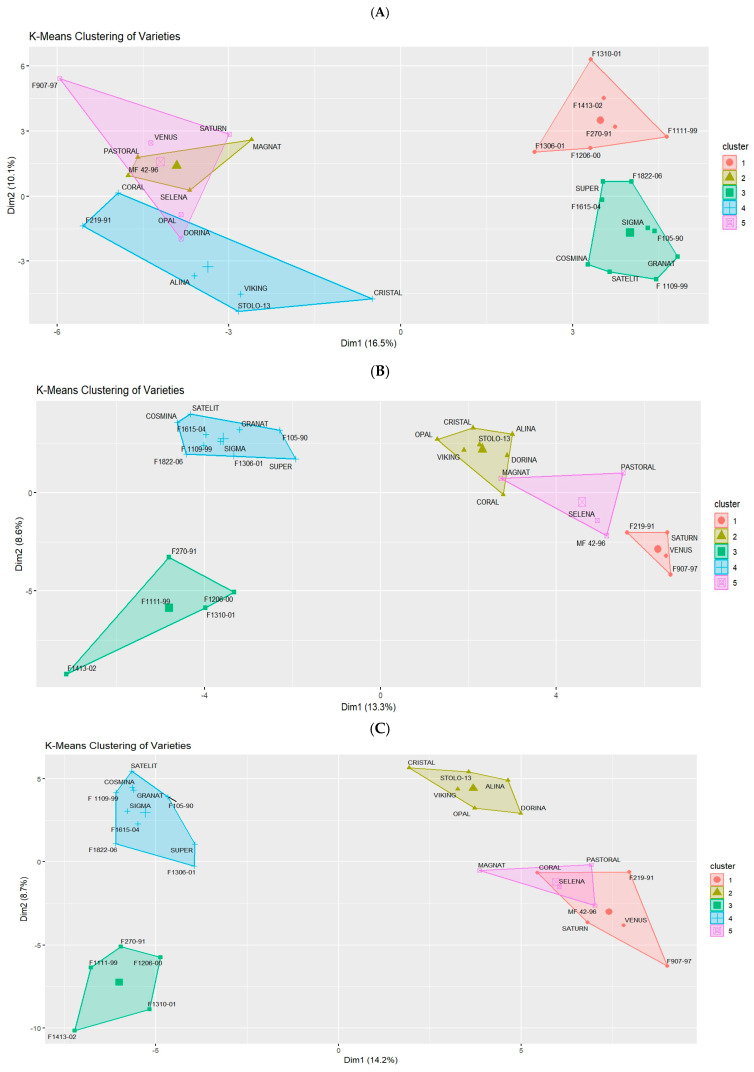
Comparative clustering of varieties using hierarchical and K-means methods based on ISSR and RAPD markers: (**A**) ISSR results, (**B**) RAPD results, (**C**) combined ISSR and RAPD analysis.

As shown in Figure 7, the varieties are grouped into five clusters, with each cluster containing several varieties. The grouping depends on the similarity coefficients determined through the analysis, whether using ISSR primers, RAPD primers, or a combination of both.

The ISSR primers grouped the varieties into five distinct clusters. This categorization further enhances our understanding of the genetic relationships among the varieties. According to our results, the varieties that comprise each cluster are as follows (Figure 6A and Figure 7A):

- *Cluster I* includes the varieties “F1206-00”, “F1306-01”, “F270-91”, “F1413-02”, “F1310-01”, and “F1111-99”.
- *Cluster II* includes the varieties “Pastoral”, “Magnat”, “Selena”, and “MF 42-96”.
- *Cluster III* includes the varieties “Satelit”, “F1109-99”, “F105-90”, “Granat”, “Cosmina”, “Sigma”, “F1615-04”, “F1822-06”, and “Super”. This cluster is notably diverse, indicating a broad spectrum of genetic traits.
- *Cluster IV* includes the varieties “Alina”, “Stolo-13”, “Viking”, “Cristal”, “F219-91”, and “Coral”.
- *Cluster V* includes the varieties “Dorina”, “Saturn”, “Opal”, “Venus”, and “F907-97”.

RAPD primers distinctly separate the clusters, with no clear overlap between them, providing a more general view of how the varieties are differentiated from one another. As a result of the analyses, the RAPD primers divided the 30 varieties into 5 clusters as follows (Figure 6B and Figure 7B):

- *Cluster I* includes the varieties “F219-91”, “Saturn”, “Venus”, and “F907-97”.
- *Cluster II* includes the varieties “Alina”, “Stolo-13”, “Viking”, “Cristal”, “Coral”, “Dorina”, and “Opal”.
- *Cluster III* includes the varieties “F1206-00”, “F270-91”, “F1413-02”, “F1310-01”, and “F1111-99”.
- *Cluster IV* includes the varieties “Satelit”, “F1109-99”, “F105-90”, “Granat”, “Cosmina”, “Sigma”, “F1615-04”, “F1306-01”, “F1822-06”, and “Super”.
- *Cluster V* includes the varieties “Pastoral”, “Magnat”, “Selena”, and “MF 42-96”.

The ISSR and RAPD primers collectively provide a comprehensive view of the genetic relationships among the 30 varieties, organizing them into five distinct clusters (Figure 7C):

- *Cluster I* includes the varieties “F219-91”, “Coral”, “Saturn”, “Venus”, and “F907-97”
- *Cluster II* includes the varieties “Alina”, “Stolo-13”, “Viking”, “Cristal”, “Dorina”, and “Opal”
- *Cluster III* includes the varieties “F1206-00”, “F270-91”, “F1413-02”, “F1310-01”, and “F1111-99”
- *Cluster IV* includes the varieties “Satelit”, “F1109-99”, “F105-90”, “Granat”, “Cosmina”, “Sigma”, “F1615-04”, “F1306-01”, “F1822-06”, and “Super”
- *Cluster V* includes the varieties “Pastoral”, “Magnat”, “Selena”, and “MF 42-96”.

Based on the analyses conducted, it can be observed that both ISSR and RAPD primers grouped the varieties into five distinct clusters, highlighting a stable differentiation in the genetic diversity of the varieties. However, there are multiple variations between the two types of primers. Although there are similarities, such as how varieties are grouped into clusters like *Cluster IV* in the RAPD molecular analysis, which is like *Cluster III* in the ISSR analysis, these results suggest that the nature of the primers determines and reveals the genetic relationships among the analyzed varieties differently. Therefore, using the most efficient primers from both groups, RAPD and ISSR, is essential to cover both aspects and provide a complementary analysis of genetic diversity. A significant consistency regarding the variation is observed, containing the same varieties (“Pastoral”, “Magnat”, “Selena”, and “MF 42-96”), revealing that these varieties are closely related and grouped into a compact cluster. This emphasizes the importance of understanding that the nature and specificity of the primers offer a perspective in selecting the most suitable primers.

ISSR primers are more specific than RAPD primers because they amplify sequences between simple repeats (microsatellites) in the genome [28,41]. Due to this, ISSR primers can detect deeper differences between populations, which RAPD primers might overlook. This may explain why, in the RAPD primer analyses (Figure 7B), the clusters are not as intercalated as they are in the ISSR primer analysis (Figure 7A).

## 3. Discussion

The similarity analyses conducted in this study, based on the Jaccard similarity coefficient obtained from the molecular analyses, allowed us to identify and demonstrate the levels of similarity among the alfalfa varieties analyzed. The results showed a high degree of similarity between certain alfalfa varieties, such as “Cristal” and “Viking” (0.81), which had the highest similarity coefficient, as well as “Stolo-13” and “Viking” (0.76). On the other hand, the variety “Pastoral” exhibited the lowest similarity coefficients when compared to other varieties such as “F1413-02” (0.39), followed by “F270-91” (0.42), “Cosmina” (0.43), “Satelit” (0.44), and “F109-99” (0.45). A similar case was observed for the variety “F1413-02”, which showed the lowest similarity with the variety “Selena” (0.39), and with most of the varieties having a similarity coefficient below 0.45.

Therefore, the varieties “Pastoral” and “F1413-02” can be considered important candidates for inducing genetic variability within the breeding program. The high similarity levels between certain varieties might be due to these varieties sharing similar genetic traits, which could be beneficial for maintaining certain desired traits [42,43,44] but not for increasing the heterosis effect [18], which is necessary for enhancing the productivity of the varieties [45]. Many authors have noted the slow progress in alfalfa variety productivity [46,47], especially considering that this crop is very useful for crop rotation, mainly due to its ability to fix atmospheric nitrogen [48,49]. Additionally, increasing the level of heterosis can positively influence this nitrogen-fixing capacity [50,51,52].

Crossing alfalfa varieties with different genetics can lead to an increase in the heterosis effect [53,54], which may result in varieties with higher performance in terms of productivity, disease tolerance, and adaptability to variable environmental conditions [53,54,55,56].

Therefore, from our results, the selection to achieve greater hybrid vigor and a higher heterosis effect could be significantly enhanced by using the “Pastoral’’ and “F1413-02” varieties in future crosses as a strategy to improve production capacity. On the other hand, avoiding the use of pairs like “Viking” and “Cristal” (0.81) and “Viking” and “Stolo-13” (0.76) in crosses might not initiate a significant level of genetic progress and thus could conserve both economic and physical resources, improving the progress of alfalfa breeding programs. In this context, the use of biotechnology in breeding, especially in alfalfa breeding programs, proves to be particularly useful [57].

Molecular markers have been demonstrated to be very effective in demonstrating genetic diversity in alfalfa varieties by several authors [30].

Following the analyses conducted, it was demonstrated that ISSR primers and RAPD primers have potential for improving molecular analyses for determining variability among the 30 alfalfa varieties examined.

The most efficient ISSR primer was A17, which generated over 31 bands, all of which were polymorphic. On the other hand, A21 was found to be the least efficient in the analyses, with a genetic diversity index of 9.17 among the alfalfa varieties. Studies suggest that the ISSR primer with the nucleotide sequence is capable of identifying polymorphism, not only in alfalfa but also in other species [58,59].

The most efficient RAPD primer was L-07, which generated 21 bands, all of which were polymorphic, with a genetic diversity index of 4.79. The L-07 primer also produced the highest number of bands in other species, such as *Puccinia triticina* [60].

The similarity degree analyses conducted helped us to improve the economic efficiency of molecular analyses. For example, in the case of primers L-12 and G-10, the similarity coefficient was high, allowing us to select only one of them to avoid the redundancy of bands generated by the primers, thereby enhancing economic efficiency.

The efficiency of detecting genetic diversity in alfalfa was better with ISSR primers compared to RAPD primers. Although a smaller number of ISSR primers was used, they appear to be more efficient and effective in analyzing genetic diversity compared to RAPD. The ISSR primers provided greater efficiency per primer and a significantly higher discrimination index of 4.20 compared to 2.10 for RAPD primers. Similar results leading to the conclusion that ISSR revealed a greater capacity in detecting genetic diversity were found in other studies in other species such as *Hordeum vulgare* L. [61], *Cocus nucifera* L. [62], triticale [63], *Brassica* [31], *Dalbergia sissoo* [64], and many others [41,65].

The analysis of the 30 alfalfa varieties showed that both ISSR and RAPD markers can detect genetic diversity. However, they provide different information, which may underscore the importance of complementary use.

Both ISSR and RAPD markers have been widely used in the study of alfalfa, a key forage crop with significant economic and ecological value. Through this methodology, breeders can identify genetically diverse lines that could be used to enhance the genetic pool available for breeding and for preservation of valuable varieties.

The complementary use of ISSR and RAPD primers has highlighted more clarity in grouping alfalfa varieties, providing further perspectives to explore the use of other types of primers, such as co-dominant ones, which have also shown good results in other studies in determining genetic diversity in alfalfa [21] and other species [66,67,68].

Other authors have also highlighted the importance of the simultaneous use of ISSR and RAPD markers to gain a more comprehensive understanding of genetic diversity, as noted by Fernandez et al. (2002) [69] and Tonk et al. (2014) [63]. Therefore, the combined use of ISSR and RAPD markers is justified and recommended to improve the accuracy and details of genetic diversity analysis, thereby supporting more informed decisions in selection and breeding within alfalfa research programs.

The use of statistical molecular analyses provides important efficiency regarding primers’ capacity. Therefore, breeding programs can improve the economic efficiency in grouping alfalfa varieties within the methodology presented, thereby enhancing the capacity and sustainability.

Another type of primer that could be used is the Simple Sequence Repeat (SSR) primer, which is co-dominant, allowing the heterozygote in diploid genomes to be distinguished [70]. However, SSR analysis detects the variation in DNA at pre-determined sequence sites, while ISSR analysis is more efficient at scanning the whole genome. These two types of marker analysis can be used to configure the polymorphism more effectively than either marker dose alone due to the nature of genetic variation detected by each marker category [71].

The comparative assessment of IRAP (Inter-Retrotransposon Amplified Polymorphism), REMAP (Retrotransposon-Microsatellite Amplified Polymorphism), ISSR (Inter-Simple Sequence Repeat), and SSR (Simple Sequence Repeat) primers conducted by Abdollahi Mandoulakani et al. (2018) demonstrated a significant reliance on ISSR markers, with ISSR primers showing the highest value in distinguishing polymorphism [29].

On the other hand, Single Nucleotide Polymorphisms (SNPs) could offer additional insights into the genetic landscape of alfalfa being used widely in alfalfa research in recent years [72,73,74]. Incorporating SNP analysis alongside ISSR and RAPD markers could improve genetic diversity analyses. While ISSR and RAPD markers are useful for scanning large regions of the genome, SNPs could provide a more granular level of detail [73]. Therefore, by integrating SNP data, researchers could achieve a more comprehensive understanding of genetic variation within alfalfa populations and make an even more accurate decision in pre-selection of the varieties suitable for new crosses.

Regarding cost-effective solutions for alfalfa breeding programs aiming to introduce molecular markers for selecting the most beneficial crosses to enhance genetic variability, ISSR primers are a logical choice used by many authors in their diversity analyses for alfalfa [75,76,77,78]. Their implementation, combined with molecular statistical analysis, can effectively increase genetic variability by pre-selecting the most distinguishable genotypes. For instance, the varieties “Pastoral” and “F1413-02” showed the greatest genetic distance compared to most other varieties. Thus, these varieties could be exploited further to enhance the heterosis effect. However, SSR primers are often used for detecting morphological and yield-related traits or analyzing relationships among different genotypes, making them valuable tools in later stages of breeding programs. The introduction of molecular markers in alfalfa breeding programs requires a well-defined strategy, particularly from an economic perspective [79].

The limitation of the study is the relatively low number of alfalfa varieties examined, although it was statistically sufficient [80]. The primers used have proven effective for this specific germplasm; however, it is important to note that they may not be equally successful across all genotypes.

Although the use of ISSR and RAPD primers in genetic diversity analyses offers certain advantages, there are associated drawbacks that could affect data analysis, particularly due to their reproducibility of the primers, especially in the case of RAPD, that can increase the genotyping errors [81].

These issues can be reduced, in principle, by using rigid laboratory protocols and conducting repeatability tests [82,83]. The purpose of these tests is to repeat the analyses and retain for further analysis only the bands that appear in both the initial and subsequent screenings [81]. Another way to reduce the level of genotyping errors caused by reproducibility of the primers used is to implement specific primers such as SSR developed in the study of Petolescu Cerasela et al., 2010, and Ioja-Boldura et al., 2010 [84,85].

Therefore, this study offers a model for breeding programs in the early stages of introducing molecular markers. We suggest starting with the most reliable and commercially available varieties that have the strongest potential to enhance heterosis.

Therefore, to optimize costs, it is advisable to select a smaller number of varieties from the alfalfa breeding program and test many primers early on. This approach will help pre-select the most beneficial primers, whether using only ISSR primers or a combination of ISSR and RAPD primers. This strategy should include an accurate understanding of the similarity groups among existing varieties, followed by careful implementation within the breeding program. Consequently, applying cost-effective strategies to select specific primers for the available germplasm in the initial stages, without testing many individuals, is necessary for any program aiming to introduce polymorphic analyses and pre-determine the best potential crosses into their alfalfa breeding efforts.

## 4. Materials and Methods

### 4.1. Plant Materials

The biological material used consisted of 30 alfalfa genotypes (*Medicago sativa* L.). The genotypes studied belong to Romanian germplasm—Satelit, Saturn, Coral, Viking, Alina, F1822-06, to Romanian and foreign germplasm—Dorina, Cristal, Stolo13, Pastoral, Selena, Magnat, F907-97, F1111-99, F1413-02, Granat, Sigma, F105-90, F1306-01, F1109-99, F270-91, F219-91, Venus, or only foreign germplasm—Super, MF 42-96, F1206-00, Cosmina, F1615-04, Opal.

### 4.2. Technical Specifications for Primer Selection and the Reduction in Genotyping Errors

Primer selection was conducted from an initial collection of 50 ISSR primers and 50 RAPD primers at the Department of Molecular Biology, Faculty of Engineering and Applied Technologies, University of Life Sciences “King Mihai I”, as part of the research contract Module I: Improving the Quality of Alfalfa Feed by Modifying Plant Architecture Using Biotechnological Methods, 2005–2008.

From the primers selected for this study, genotyping errors were found to be 0.5–1.8% for ISSR and 4.2–7.3% for RAPD depending on the primers selected. Furthermore, to minimize analysis errors, only the bands that appeared in both the initial and subsequent screenings were considered for both RAPD and ISSR primers [81]. The evaluation of the genotyping error of the primers was conducted using the method proposed by Bonin et al. (2004) [82], following the suggestions of these authors to minimize errors.

In order to minimize genotyping errors, DNA samples from pure *Medicago sativa* L. plants were selected, thereby eliminating potential impurities linked to genetic variability among the alfalfa varieties. SSR primers developed in previous studies (Petolescu Cerasela et al., 2010 and Ioja-Boldura et al., 2010) were also were used for the selection [84,85].

Additionally, the primers were chosen based on their reproducibility in *Medicago sativa* L. [86,87], as their reproducibility had been tested in other species as well [88,89,90,91]. To ensure the accuracy of selection for both ISSR and RAPD genotyping, all reactions were performed in three independent repetitions for each DNA sample to select the primers with the best reproducibility in *Medicago sativa* L.

### 4.3. Molecular Analyses and Correlations between Primers Assays

We used molecular techniques of genetic variability detection (extraction of genomic ADN, enzymatic amplification by PCR reaction using ISSR and RAPD markers, and analysis of the reaction products by agarose gel electrophoresis) and statistical–mathematical methods of result processing and interpretation [92,93].

The correlation similarity coefficient matrix between primers was applied to identify primers with either clear distinctiveness or excessive redundancy. This approach allowed for the selection of the most efficient primers by eliminating redundant primers across the 30 analyzed varieties.

The correlation coefficient values were analyzed among different ISSR and RAPD markers. In cases of positive correlations, when the band weight values increase similarly with another primer across the 30 analyzed varieties, it indicates that the pair of primers targets similar regions of the genome and produces the same patterns. Consequently, in cases of strong correlations (0.7–0.8) or very strong correlations (0.9–1.0), the primers are considered highly redundant, and it is advisable to use only one of the analyzed pairs. On the other hand, primers with very weak correlations (<0.1) or weak correlations (0.2–0.3) are likely to complement each other, making them suitable for combined use. In the best scenario, when the correlation coefficient is significantly negative, it suggests a high complementarity between the two primers [94].

### 4.4. Genomic DNA Extraction and ISSR and RAPD Assays

Fragments from the fresh leaf samples resulting from the plants selected were used for the extraction of genomic DNA. The Maxwell™ 16 Instrument from Promega was used for the automatic extraction of nucleic acids. This device is designed for the extraction and purification of nucleic acids from any type of tissue, whether plant or animal. It requires 50 mg of plant tissue, uses kits, and can process up to 16 samples in 42 min. DNA of 99% purity is obtained, which is used in a wide range of applications.

Additionally, DNA concentration and quality were evaluated with the Nanodrop 8000 (ThermoFisher Scientific, Waltham, MA, USA). The concentration was diluted to 100 ng/µL in all samples.

### 4.5. Enzymatic Amplification by PCR Reaction

A standard PCR reaction is performed in a volume of 25 µL containing: polymerase, dNTPs, MgCl_2_, primers, nuclease-free distilled water, and the DNA template that contains the sequence to be amplified. The reaction mixture is made once all samples are amplified, after which it is distributed into 0.2 mL Eppendorf tubes. These operations are carried out in a mini-PCR cabinet, on ice, to avoid any issues related to incorrect handling. To evaluate the intergenotypic variability in the alfalfa studied genotypes, RAPD and ISSR markers are used. The reaction mixtures and amplification conditions were different, depending on the type of primer used. The thermocycler used was a Corbett type. Non-DNA control samples were included in every run of the experiment to ensure accuracy and to evaluate any potential contamination.

For the PCR reaction, the mixture intended for obtaining the amplification products was made up of the following:

1. GoTaq^®^ Green Master Mix 2x kit (Promega, Madison, WI, USA) which includes: GoTaq^®^ DNA
2. Primer: 10 pmol/µL (Fermentas, Waltham, MA, USA);
3. 100 ng/µL genomic DNA.
4. Nuclease-free distilled water.

### 4.6. Reaction Conditions for RAPD Markers

The conditions under which amplification was performed were as follows:

- initial DNA denaturation: 4 min at 94 °C, followed by 45 cycles, each cycle having the following stages:
  - denaturation: 3 min, at 94 °C;
  - primer annealing: 1 min at 36 °C;
  - extension: 2 min at 72 °C.
- DNA synthesis: 3 min at 72 °C

At the end, the samples were cooled to 4 °C in the thermocycler and stored in the refrigerator until use.

### 4.7. Reaction Conditions for ISSR Markers

The conditions under which the amplification was carried out were as follows:

- initial DNA denaturation: 5 min at 94 °C, followed by 35 cycles, each cycle having the following stages:
  - denaturation: 1 min, at 94 °C;
  - primer annealing: 1 min at 46–51 °C;
  - extension: 3 min at 72 °C.
- DNA synthesis: 7 min at 72 °C

At the end, the samples were cooled to 4 °C in the thermocycler and stored in the refrigerator until use.

### 4.8. Analysis of Reaction Products by Agarose Gel Electrophoresis

The migration of DNA in the gel occurs using a voltage between 80 and 100 V. The DNA in the gel was visualized with the help of a UV transilluminator and was photographed. The molecular weight markers utilized included the following PCR markers: 1000, 750, 500, 300, 150, and 50 base pairs.

### 4.9. Mathematical and Statistical Methods for Results Interpretation

In the calculations made following the analyses conducted with various primers, only the distinct bands were recorded as present (1), while the bands that exhibited very low resolution were marked as absent (0). These bands were then encoded into a binary matrix.

To characterize the potential of different molecular marker systems to assess the inter-population variability in alfalfa genotypes, different parameters were calculated in Rstudio v4.4.1 as follows:

- the total polymorphism generated by a specific primer (PIC) which indicates its discriminatory power [95]:
  (1) $$PIC=1-\sum_{i=1}^{n}P_{ij}^{2}-\sum_{i=1}^{n-1}\sum_{j=i+1}^{n}2P_{i}^{2}P_{j}^{2}$$
- Pi—the frequency of allele i; Pj—the frequency of allele j; Pij—the frequency of allele i for locus j; n—the total number of loci.
- the discrimination index (PI), which attests to the efficiency of a specific primer in detecting polymorphism [96].
  (2) PI=∑PIC
- the marker index (MI) was used as a measure of the overall utility of a marker in polymorphism detection, considering both the number of polymorphic loci (EMR) and the PIC value [41,97]:
  *MI = EMR* ∗ *PIC* (3)
- EMR = np (np/n); np is the number of polymorphic loci and n is the total number of loci.
- Rp (resolving power) assesses the ability of a primer to distinguish between different genotypes [41,97]:
  (4) $$Rp=\sum_{i=1}^{np}Ib$$
- I*_bi_* is the band informativeness, calculated as 2 × ∣0.5−*p_i_*∣, where *p_i_* represents the band presence ratio.

Genetic similarity among the analyzed alfalfa varieties was determined using the Jaccard coefficient based on data obtained from ISSR and RAPD primers. K-means clustering was then performed using RStudio v4.4.1 to categorize the alfalfa varieties into distinct groups based on genetic similarity, with clustering conducted separately for ISSR, RAPD, and combined ISSR and RAPD data. Additionally, a multivariate heatmap illustrating the genetic diversity of the 30 alfalfa varieties was generated using the heatmap module in RStudio v4.4.1 Hierarchical clustering was conducted to further explore the genetic relationships among the alfalfa varieties using RStudio v4.4.1, with bootstrap analysis applied to assess the stability of the clusters. The bootstrap values, indicated on the branches, provide a measure of confidence for each cluster, with higher values suggesting more reliable groupings [23].

## 5. Conclusions

Following the study conducted on the 30 alfalfa varieties, we were able to distinguish the similarities between the varieties successfully using ISSR and RAPD primers. The analyses highlighted that the best primer among the ISSR primers was A17, which generated 31 bands and had a genetic diversity index of 9.17 among the alfalfa varieties. On the other hand, in the case of the RAPD primers, the most efficient primer was L-07, with an efficiency of 4.79.

Overall, the ISSR primers proved to be more efficient compared to RAPD primers. However, it is important to mention that their simultaneous use resulted in a more effective and clearer distribution of varieties. The statistical analyses demonstrated efficiency by eliminating similar primers, such as G10 and G19, which showed a very high correlation index. Therefore, molecular statistical analysis is necessary for better economic efficiency in alfalfa breeding programs.

The utility of molecular markers, specifically primers, must be tailored to the germplasm used, enabling breeders to identify which varieties are genetically more distinct or closely related, thus allowing them to make more specific decisions in their breeding programs.

## Figures and Tables

**Table 1:** Polymorphism rate for the alfalfa genotypes using ISSR primers.

No. Crt.	Primer	Primer Sequence	Band Size (pb)	Total Bands (n)	Polymorphic Bands (np)	Pb(%)	PIC	MI	Rp
1	A12	(GA)6CC	220–1650	25	20	80.00	0.20 ± 0.13	3.23 ± 0.18	7.53
2	A13	(GT)6CC	150–1510	27	19	70.37	0.24 ± 0.09	3.24 ± 0.17	7.66
3	A17	(GTG)3GC	240–2090	31	31	100.00	0.30 ± 0.07	9.17 ± 0.10	16.80
4	A21	(CA)6AC	280–1320	15	9	60.00	0.26 ± 0.11	1.41 ± 0.20	4.46
5	UBC818	(CA)7G	300–2120	19	16	84.21	0.29 ± 0.09	3.94 ± 0.18	8.73
6	Mean	ISSR primers	150–2120	23.40	19.00	78.91	0.25 ± 0.04	4.19 ± 2.93	9.03

Note: n: Total number of bands; np: Number of polymorphic bands; Pb%: polymorphic bands percentage; PIC: Polymorphic Information Content; MI: Marker Index (MI = EMR ∗ PIC), EMR = np (np/n); Rp (resolving power) = $\sum_{i=1}^{np}Ib$i, where np represents the number of polymorphic locus and Ibi is the band index for each band.

**Table 2:** Polymorphism rate for the alfalfa genotypes using RAPD primers.

No. Crt.	Primer	Primer Sequence	Band Size (pb)	Total Bands (n)	Polymorphic Bands (np)	Pb(%)	PIC	MI	Rp
1	B-07	GGTGACGCAG	190–1250	9	8	88.89	0.16 ± 0.11	1.14 ± 0.15	2.06
2	G-03	GAGCCCTCCA	320–900	7	4	57.14	0.11 ± 0.07	0.25 ± 0.09	0.53
3	G-04	AGCGTGTCTG	300–1250	12	10	83.33	0.22 ± 0.10	1.81 ± 0.16	3.33
4	G-06	GTGCCTAACC	220–1000	10	7	70.00	0.25 ± 0.11	1.22 ± 0.19	3.00
5	G-10	AGGGCCGTCT	300–1500	15	12	80.00	0.17 ± 0.09	1.62 ± 0.14	3.00
6	G-16	AGCGTCCTCC	300–1000	11	9	81.81	0.25 ± 0.12	1.83 ± 0.19	4.06
7	G-17	ACGACCGACA	180–1100	14	11	78.57	0.23 ± 0.11	1.99 ± 0.18	4.46
8	G-18	GGCTCATGTG	190–1250	12	11	91.66	0.18 ± 0.10	1.90 ± 0.14	3.00
9	G-19	GTCAGGGCAA	270–1350	13	13	100.00	0.26 ± 0.12	3.46 ± 0.17	6.66
10	L-03	CCAGCAGCTT	260–750	10	8	80.00	0.18 ± 0.09	1.17 ± 0.14	2.13
11	L-07	AGGCGGGAAC	170–1590	21	21	100.00	0.22 ± 0.10	4.79 ± 0.15	8.33
12	L-12	GGGCGGTACT	280–1300	15	15	100.00	0.25 ± 0.15	3.13 ± 0.14	5.33
13	L-14	GTGACAGGCT	230–1130	11	11	100.00	0.27 ± 0.11	3.01 ± 0.16	5.87
14	Mean	RAPD primers	170–1590	12.30	10.76	85.49	0.21 ± 0.04	2.10 ± 1.20	3.98

Note: n: Total number of bands; np: Number of polymorphic bands; Pb%: polymorphic bands percentage; PIC: Polymorphic Information Content; MI: Marker Index (MI = EMR ∗ PIC), EMR = np (np/n); Rp (resolving power) = $\sum_{i=1}^{np}Ib$i, where np represents the number of polymorphic locus and Ibi is the band index for each band.

**Table 3:** Comparative analysis of banding patterns generated by RAPD and ISSR markers.

Parameter	ISSR	RAPD
Number of primers	5	13
Total number of bands	117	160
Number of bands/primers	23.40	12.30
Number of polymorphic bands	95.00	140.00
Polymorphism rate (%)	81.19	87.50
Analysis efficiency index	4.20	2.10
Polymorphism/primer	0.811	0.874
Discrimination index	9.03	3.98
Size of bands (bp)	150–2120	170–1590

**Table 4:** Specific alleles for certain alfalfa genotypes.

No.	Genotype	Specific Alleles	No.	Genotype	Specific Alleles
1	Pastoral	A 12–1320	11	Stolo 13	G 03–500
2	Pastoral	G 18–1000	12	Stolo 13	G 18–1250
3	MF 42-96	L 07–540	13	Sigma	A 12–490
4	MF 42-96	L 07–580	14	Cosmina	A 12–744
5	F 1111-99	G 04–600	15	Granat	A 21–780
6	F 1111-99	G 06–1000	16	F 907-97	A 21–1150
7	F 1306-01	A 12–300	17	Saturn	G 10–350
8	F 1306-01	G 10–650	18	Dorina	G 10–1300
9	F 1206-00	G 17–1100	19	Coral	L 14–1000
10	F 1206-00	G 19–900	20	F 1413-02	L 12–450

## Data Availability

The data are included in the article.
